# Targeting Executive Function and Language Impairments with tACS Combined with Behavioral Intervention in Primary Progressive Aphasia: A Case-Series, Pilot Investigation

**DOI:** 10.3390/brainsci15111199

**Published:** 2025-11-07

**Authors:** Kyriaki Neophytou, Dimitrios S. Kasselimis, Georgia Angelopoulou, Areti Deligiannaki, Rafailia Bourtsoukli, Eleni Peristeri, Vasilina Spanou, Sokratis G. Papageorgiou, Vasilios C. Constantinides, Constantin Potagas, Kyrana Tsapkini

**Affiliations:** 1 Department of Neurology, Johns Hopkins Medicine, 600 N. Wolfe Street, Phipps 446, Baltimore, MD 21287, USA; 2 Department of Social Sciences, School of Humanities and Social Sciences, University of Nicosia, 2417 Nicosia, Cyprus; 3 Department of Psychology, Panteion University of Social and Political Sciences, 176 71 Athens, Greecerafaeliabourt@gmail.com (R.B.); 4 Neuropsychology and Language Disorders Unit, 1st Department of Neurology, School of Medicine, National and Kapodistrian University of Athens, Eginition Hospital, 115 28 Athens, Greece; 5 Department of Theoretical & Applied Linguistics, School of English, Aristotle University of Thessaloniki, 546 36 Thessaloniki, Greece; eperiste@enl.auth.gr (E.P.);; 6 1st Department of Neurology, National and Kapodistrian University of Athens, Eginition Hospital, 115 28 Athens, Greecevassilis.kon@hotmail.com (V.C.C.); 7 Department of Cognitive Science, Johns Hopkins University, Baltimore, MD 21218, USA

**Keywords:** executive functions, language, primary progressive aphasia (PPA), transcranial alternating current stimulation (tACS), alpha rhythm, computerized cognitive training (CCT)

## Abstract

**Background/Objectives**: Executive function (EF) impairments are found in a variety of neurodegenerative disorders, including in Primary Progressive Aphasia (PPA), which is primarily characterized by language impairments. The goal of this preliminary investigation was to evaluate the hypothesis that, by targeting domain-general EFs, domain-specific functions—specifically, language processing—might also be improved in this population. **Methods**: This case series included four Greek-speaking individuals with PPA who underwent behavioral and neurostimulation treatment daily for 15 consecutive sessions. Behavioral treatment was performed through Computerized Cognitive Training (CCT) that targeted various EF functions. Neurostimulation treatment included alpha-rhythm transcranial alternating current stimulation (tACS) over the left dorsolateral prefrontal cortex (DLPFC), previously implicated in EF functioning. EF and language performance was assessed before (pre-) and after (post-) treatment and was also compared against the performance of healthy control individuals. **Results**: The pre- to post-treatment comparisons showed improvements primarily in EF functions, with heterogeneous improvements in language functions across the four cases. Except for one task (N-back), in which all four patients showed numerical improvement, the pattern of numerical gains differed across patients. **Conclusions**: While the treatment protocol targeted EF functioning, improvements were found for both EF and language processes (albeit more variable across patients). These results support the hypothesis that improvement in domain-general functions may lead to improvements in domain-specific functions as well. These preliminary findings can be used as guiding evidence for the design of future, large-scale clinical trials that will allow us to generalize conclusions to the broader PPA population.

## 1. Introduction

‘Executive functions’ (EFs) is an umbrella term for a set of high-level processes, such as inhibition, selective attention, and working memory, that we recruit during several—usually challenging—tasks [1]. EF impairments are prevalent in a variety of neurodegenerative disorders, independently of what the primary impairment is. For example, EF deficits have been reported in neurodegenerative disorders that mostly affect memory, such as Mild Cognitive Impairment [2] and Alzheimer’s Disease (AD) [3], as well as in disorders that are primarily characterized by language impairments, such as Primary Progressive Aphasia (PPA) [4,5,6]. Thus, finding treatments for EF impairments can be beneficial across a variety of populations.

The involvement of prefrontal cortical regions in EF [7] has led to extensive investigations of the effectiveness of neurostimulation for EF when applied in the dorsolateral prefrontal cortex (DLPFC). Meta-analyses have shown that tDCS and repetitive transcranial magnetic stimulation (rTMS) over the DLPFC lead to improved performance in EF tasks in healthy individuals as well as in clinical populations, such as those with AD, post-stroke aphasia, and behavioral variant frontotemporal dementia (bvFTD) [8,9,10]. A handful of studies have also investigated the effects of DLPFC stimulation in PPA and have shown positive effects on language performance, specifically for naming [11,12,13].

Beyond the neuroanatomical correlates of EF, various studies have also tried to uncover the electrophysiological underpinnings of EF. The alpha rhythm, which most often refers to activity in the 8–12 Hz range, has been associated with a variety of cognitive functions of the executive system, such as selective attention [14], top-down inhibitory control processes [15], and working memory [16]. A prominent reduction in alpha wave activity has also been reported in patient populations with EF deficits, such as bvFTD [17], as well as AD and Mild Cognitive Impairment (MCI) [18]. Together, these findings highlight the importance of alpha rhythm in EF.

Neuromodulation techniques, such as transcranial alternating current stimulation (tACS), can capitalize on this information to target brain activity not only in specific brain regions but also at specific frequencies. tACS is a simple, non-invasive technique that can be used to manipulate neural rhythmicity. Specifically, low-intensity electric current (typically between 1 and 4 mA) is delivered to the scalp by applying two or more electrodes connected to a battery-powered current stimulator. As the name suggests, the delivered electrical current is alternating, meaning that the current has a sinusoidal waveform in which the voltage changes gradually from positive to negative every half-cycle [19]. tACS is applied at conventional EEG frequencies (i.e., 0.1–80 Hz), and it is therefore believed to interact with the existing rhythms on the brain cortex [20]. Specifically, tACS is thought to entrain neuronal networks, which means that the ongoing brain activity becomes temporally aligned with the external alternating current oscillation. The efficacy of tACS is emerging across conditions, for cognitive functioning in general [21], as well as for specific EF processes, including cognitive control in MCI [22] and attention [23] and decision-making [24] in healthy controls. Importantly, tACS has also been shown to have a greater effect than tDCS on a variety of EF processes, such as cognitive control [25], associative memory [26], as well as working memory [27].

Another line of research with important implications for treatment interventions is evidence showing that the recruitment of EF processes that are considered to be domain-general functions can partially compensate for focal disruptions of specialized cognitive functions, such as language [28]. Based on this, we can hypothesize that, by targeting domain-general EF functions, domain-specific functions might also benefit. A suitable population to test this hypothesis is PPA. PPA is an age-related degenerative neurological syndrome, mainly characterized by a gradual deterioration of language functions [29]. In most classifications, PPA is divided into three subtypes or variants, each associated with distinct regions of brain atrophy, diverse pathologies, as well as with diverse neuropsychological profiles [30]. These are non-fluent (nfvPPA), semantic (svPPA), and logopenic (lvPPA) variant PPA.

While language impairments are the first and most prominent behavioral symptom, more recently, there has been increasing interest in EF in PPA as well [4,5,6]. Conflicting results have been reported regarding EF impairments in PPA. However, a recent meta-analysis has shown that individuals with PPA show poorer EF skills than healthy control individuals [5], often with differences across PPA variants [4,31]. The prominence of EF impairments in PPA has been reported cross-linguistically. Of particular relevance to the current study are recent findings that showed worse EF performance for Greek-speaking lvPPA and nfvPPA individuals than healthy control individuals and a strong relationship between their EF and language performance [32,33].

Given the prominence of language impairments in PPA, the majority of neuromodulation studies have focused on language performance. Using tDCS, most PPA interventions have targeted the left Inferior Frontal Gyrus (IFG) and the Supramarginal Gyrus (SMG), showing significant effects across language abilities [34,35,36,37,38,39]. The current study takes this line of research a step further and investigates the hypothesis that, by targeting EF functions, language processing might also improve in this population. Motivation for this research also comes from the literature on post-stroke aphasia treatment, in which various studies have targeted EF functions, such as working memory, to improve communication skills [40,41]. This is a preliminary investigation involving a case series of four Greek-speaking patients with PPA who underwent tACS combined with behavioral treatment targeting EF. To the best of our knowledge, this is the first study investigating the effects of tACS on EF in PPA and the first study investigating the effects of tACS in PPA in Greek-speaking individuals, in general. This pilot study aims to establish the feasibility and tolerability of the protocol we used and to guide the design of future, large-scale clinical trials.

## 2. Materials and Methods

### 2.1. Participants

**Figure 1:**
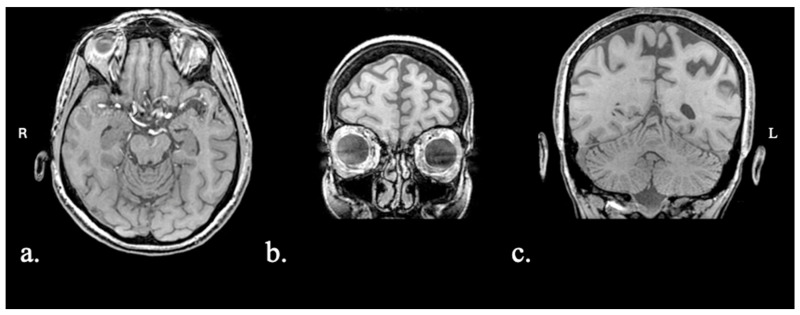
Patient FAY MRI scan: (**a**) axial view with asymmetrical (L > R) temporal atrophy; (**b**) coronal view with asymmetrical (L > R) frontal atrophy; (**c**) coronal view with asymmetrical (L > R) parietal atrophy (Scheltens 1, bilaterally).

**Figure 2:**
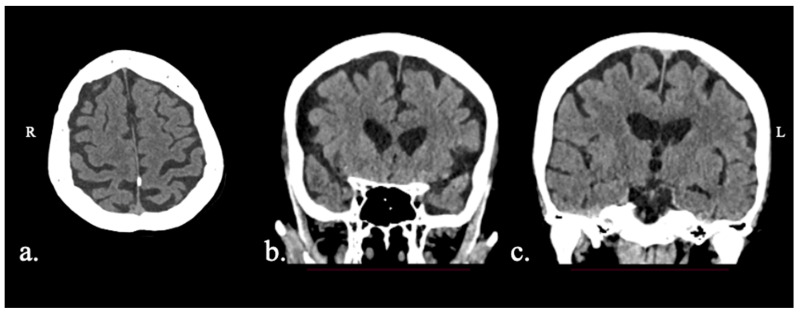
Patient XTY CT scan: (**a**) axial view with biparietal (L > R) atrophy; (**b**) coronal view with bilateral symmetric frontal atrophy; (**c**) coronal view with mild left hippocampal atrophy (Scheltens 1).

**Figure 3:**
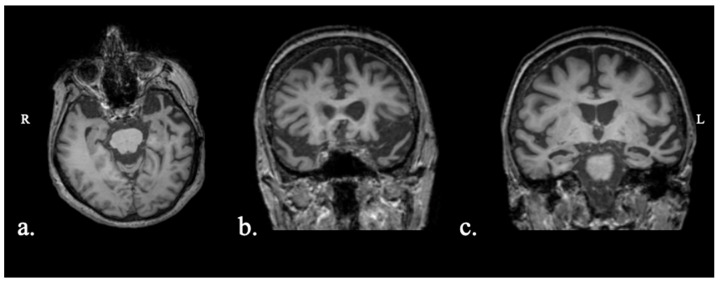
Patient ADY MRI scan: (**a**) axial view with bilateral temporal pole atrophy (L > R), with knife-edge appearance of left temporal pole gyri; (**b**) coronal view with pronounced bilateral temporal pole atrophy; (**c**) coronal view with moderate hippocampal atrophy bilaterally (Scheltens L: 3; Scheltens R: 2).

**Figure 4:**
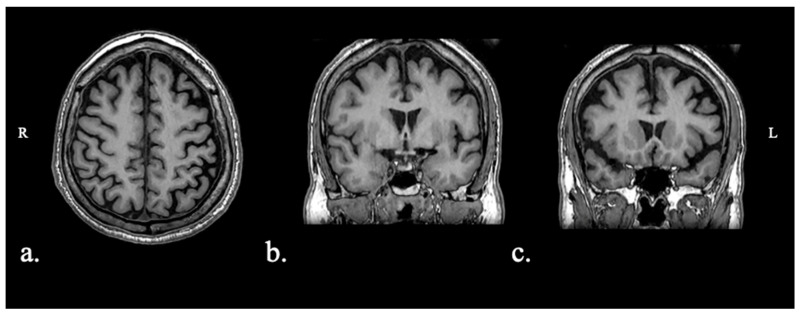
Patient IZS MRI scan: (**a**) axial view with slightly asymmetrical (L > R) parietal atrophy; (**b**) coronal view with bilateral atrophy of amygdala; (**c**) coronal view with asymmetrical (L > R) frontal atrophy.

Participants were four Greek-speaking individuals with PPA (see Table 1). PPA and variant diagnosis was performed by a team of expert neuropsychologists (DSK, GA, AD, RB) and neurologists (VCC, CP), as described in previous work [42,43]. Background language testing included the Greek version of the Mini-Linguistic State Examination [44]; the short form of the Boston Diagnostic Aphasia Examination, standardized in Greek [45]; the short forms of the Boston Naming Test and the Peabody Picture Vocabulary Test—Revised, standardized in Greek [46]; Comprehension of Instructions in Greek [47]; and two reading fluency tasks [48]. Detailed information for each case is provided below, as well as images showing their primary regions of atrophy (Figure 1, Figure 2, Figure 3 and Figure 4). Participants were recruited from the First Neurological Clinic, National and Kapodistrian University of Athens, Eginition Hospital, Athens, Greece (research protocol approval ID: 91ΩΔ46Ψ8Ν2-8ΝΕ, April 2025). Informed consent was obtained from all participants prior to participation according to the Eginition Hospital Ethics Committee.

### 2.1.1. Patient FAY

Patient FAY was a 63-year-old female. The patient presented with a 1-year history of progressive naming difficulties. MRI showed asymmetrical, left-greater-than-right, atrophy across the frontal, temporal, and parietal regions (Figure 1). Biomarker testing was not performed. The patient fulfilled criteria for logopenic variant PPA.

### 2.1.2. Patient XTY

Patient XTY was a 76-year-old female. The patient presented with a 2-year history of progressive naming difficulties. The patient did not report any other cognitive deficits, and her everyday functionality was intact. MRI was not available. Brain CT exhibited biparietal, yet asymmetrical (left > right) atrophy, bilateral symmetric frontal atrophy, and mild left hippocampal atrophy (Figure 2). Biomarker testing was not performed. The patient fulfilled diagnostic criteria for logopenic variant PPA, most probably due to underlying AD pathology.

### 2.1.3. Patient ADY

Patient ADY was a 76-year-old female. The patient presented with a 3-year history of speech difficulties, which included both naming and single-word comprehension difficulties. Over the past year she exhibited mild behavioral symptoms, with preserved everyday functionality. MRI showed asymmetrical, left-greater-than-right, temporal pole atrophy (Figure 3). Biomarker testing showed elevated total Tau and Phospho-Tau, but well-above-normal Aβ42, consistent with a non-AD pathology. The patient fulfilled criteria for semantic variant PPA, with a non-AD underlying pathology.

### 2.1.4. Patient IZS

Patient IZS was a 60-year-old male. MRI showed atrophy in the amygdala bilaterally, as well as asymmetrical (left > right) frontal and, to a lesser extent, parietal atrophy (Figure 4). Biomarker testing showed elevated total Tau and Phospho-Tau, normal Aβ42, but low Aβ42/Aβ40, consistent with AD pathology. The patient fulfilled criteria for logopenic variant PPA.

### 2.2. Treatment Protocol

Treatment was performed daily for 15 consecutive sessions (5 sessions × 3 weeks) and comprised a behavioral and a neurostimulation component as described below. Each session lasted for 40 min. All participants completed all 15 sessions.

#### 2.2.1. Behavioral

The behavioral treatment was performed through Computerized Cognitive Training (CCT), which meta-analyses have found to be effective [49,50,51]. For the purpose of this study, we focused on executive function training and used a computer-aided cognitive training program, called BrainHQ (Posit Science, 2015). BrainHQ includes tasks that target a variety of cognitive functions. BrainHQ allows for personalized training since the level of difficulty of each task is automatically adjusted for each participant through specific algorithms to maximize training gains. Specifically, difficulty is adjusted automatically based on the participant’s performance, with an increase in difficulty triggered when the user is >80% accurate and a decrease in difficulty when the user is <80% accurate. Each task lasted approximately 6–7 min, totaling 40 min of behavioral intervention per session. The efficacy of BrainHQ training has been shown across populations, including those with mild cognitive impairment [52,53,54,55], fronto-temporal dementia [10], traumatic brain injury [56,57], and healthy aging [58,59,60,61]. For this study, participants were trained in the following tasks: (1) Double Decision, (2) Freeze frame, (3) Face to Face, (4) Juggle Factor, (5) Divided Attention, and (6) Mental Map.

#### 2.2.2. Neuromodulation

Active tACS was delivered over the left DLPFC, using Soterix Transcranial Electrical Current Stimulator Clinical Trials Model 1 × 1 in an open-label design. Specifically, the anode electrode was positioned at F3 according to the 10–20 system, as is the common practice in relevant studies [10,13], and the cathode electrode was placed over the right buccinator muscle as in prior studies [35,62]. Current was delivered at 10 Hz (i.e., the average alpha band frequency) and at 2 mA for 20 min (30 s ramping), starting concurrently with behavioral intervention. In other words, during the first 20 min of the behavioral intervention, tACS was also applied. Participants reported itching at the electrode sites (particularly during ramp-up), in about one third of their sessions. The itching resolved within the first two minutes of stimulation, while one participant repeatedly reported fatigue after the end of the sessions. No other adverse effects were reported. No sessions were discontinued or missed.

### 2.3. Executive Function and Language Assessment

A set of EF and language tasks were used to evaluate the efficacy of the treatment protocol. To achieve this, performance on these tasks was assessed at two timepoints: before (pre-) and after (post-) treatment. Pre-assessments were completed within five days prior to the first treatment and post-assessments within 24 h after the final treatment. Patient performance was compared against age- and education-matched healthy control individuals.

#### 2.3.1. Executive Function Tasks

1. Trail Making Test (TMT): Draw a line between 24 consecutive circles that are randomly arranged on a page to connect numbers in increasing order (TMT-A) or to connect numbers and letters in increasing order (TMT-B). Time (in seconds) to task completion is measured. Administration and scoring are as in [63].
2. Digit and Spatial Spans (Forward and Backward): Report numbers [64] and locations (i.e., the Corsi block-tapping task [65]) in forward (FW) and backward (BW) order. Administration and scoring are as in [66,67] for the Digit Span and Corsi block-tapping task, respectively.
3. N-back: Determine if the current stimulus matches the one shown ‘*n*’ trials ago (*n* = 1). D-prime—a measurement of signal sensitivity that takes into account hits and false alarms—is calculated.

#### 2.3.2. Language Tasks

1. Spoken Naming: Name objects shown on black-and-white pictures (*n* = 30). Total word accuracy is measured. (Note: While performance was evaluated on 30 items, the available normative data differed with respect to two items. Therefore, normative data (z-scores and percentiles) are in reference to the 28 items that the two lists had in common.).
2. Picture Description: Produce a narrative based on the Cookie Theft Picture [68]. No time restrictions were applied (i.e., participants were free to speak for as long as they wanted). Proportion of nouns and verbs relative to the total number of words produced is calculated.
3. Semantic and Phonemic Fluency: Name as many items as possible that belong to a given semantic category (animals, fruits, and objects) or that start with a specific letter (Χ, Σ, A) [69]. The total number of items produced across the three prompts (per fluency type) is calculated.

### 2.4. Statistical Analysis

Performance on each task listed above, separately for pre- and post-treatment, was compared against performance from healthy, age-matched and education-matched control individuals using the Crawford–Garthwaite Bayesian test for single-case analysis [70]. Specifically, we used the test as implemented in the *crawford.test* function from the ‘psycho’ package in R (version 4.3.1) [71,72]. This test uses a non-informative prior and is specifically designed for comparisons between a single observation and a small normative sample. It provides an estimate of the probability that a randomly chosen control would obtain a lower score than the case. Effect sizes, specifically, Cohen’s d values and percentile scores, were also calculated based on comparisons with the healthy control individuals, separately for every person at each of the two timepoints.

All three analyses (Crawford–Garthwaite Bayesian tests, Cohen’s d values, and percentile scores) used the same groups of healthy control individuals. However, different healthy control groups were used to evaluate the performance of the four patients across tasks, depending on data availability. The healthy control groups varied in size: TMT (*N* = 31–32; the total number of participants for the healthy control group for TMT varied depending on the age), N-back (*N* = 21), Spoken Naming (*N* = 27), Picture Description (*N* = 35), Semantic and Phonemic Fluency (*N* = 15). Information on the demographics (i.e., age and education) of the control groups can be found in the Supplementary Material (Table S1). For Digit and Spatial Spans, only percentile normative data were available, which did not allow us to perform the Crawford–Garthwaite Bayesian tests.

For Spoken Naming and Picture Description, the age of two of our patients (XTY and ADY) was more than 2 SDs from the average age of the healthy control group. Since, for those two tasks, normative data were available at the individual level, we also performed linear regression analyses to evaluate performance relative to controls (i.e., in addition to the Crawford–Garthwaite Bayesian tests). Since normative data were not available at the individual level for all tasks, we could not perform linear regression analyses for all of them. Prior research has shown that linear regression models are a valid tool for comparing single cases with control groups [73]. A regression analysis was performed for every patient, for each of the two timepoints (pre- and post-treatment), with task score and Group (healthy control versus patient) as the dependent and independent variables, respectively. Age and education were used as covariates.

## 3. Results

**Figure 5:**
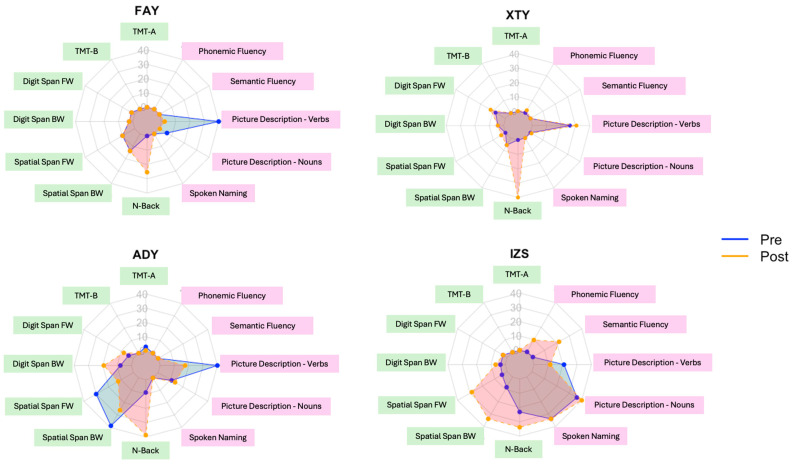
Percentile scores for individual EF and language performance per task. Tasks in green are EF tasks, and tasks in pink are language tasks. Note: percentile scores are capped at the 50th percentile for illustration purposes.

The performance on each task and the associated z-values (based on comparisons with available norms from healthy, age-matched control individuals) are presented in Table 2. Figure 5 presents percentile scores per individual per task. For instance, for the N-back task, in all four patients, post-treatment performance was at a higher percentile than pre-treatment performance. Hence, the orange line (post-treatment performance) extends beyond the blue line (pre-treatment performance). Results from the Crawford–Garthwaite Bayesian tests, along with the associated Cohen’s d values are presented in Table 3.

The linear regression analyses for Spoken Naming and Picture Description, mostly replicated the Crawford–Garthwaite Bayesian tests findings. Specifically, patient performance was significantly worse than that of controls for all patients (both pre- and post-treatment) at *p* > 0.001 except for patient ISZ. With respect to Picture Description, for the percentage of nouns, statistically or marginally significant effects were found for patient XTY both pre- (*p* = 0.020) and post-treatment (*p* = 0.066) and for patient FAY post-treatment (*p* = 0.022). For the percentage of verbs in the Picture Description, no significant differences were found between the healthy control group and the patients—the marginally significant effect found for patient FAY post-treatment in the Crawford–Garthwaite Bayesian test was no longer statistically or marginally significant.

In sum, as shown in Table 2 and Figure 5, out of the twelve metrics (seven executive function and five language metrics), patient FAY showed numerical improvement in four metrics: two EF metrics (TMT-A and N-back), as well as two language metrics (Sematic Fluency and Spoken Naming). Patient XTY showed numerical improvement in eight metrics: four EF metrics (TMT-A, Digit Span FW, Spatial Span FW, and N-back), as well as four language metrics (Picture Description—percentage of nouns and verbs, Sematic and Phonemic Fluency). Patient ADY showed numerical improvement in four metrics: three numerical EF metrics (Digit Span FW, Digit Span BW, and N-back), as well as one language metric (Picture Description—percentage of nouns). Finally, IZS showed numerical improvement in ten metrics: all metrics, except for one EF metric (TMT-A) and one language metric (Picture Description—percentage of verbs).

Despite the numerical improvements across metrics that we observed in terms of raw scores (Table 2) and percentiles (Figure 5), it was also important to evaluate whether performance post-treatment was different relative to that pre-treatment, as compared with healthy controls (Table 3). In most cases, pre-treatment performance that was statistically significantly worse than that of healthy controls remained as such post-treatment. Nonetheless, the effect sizes values (i.e., Cohen’s d) showed changes in the expected direction: in most cases, negative values became less negative post-treatment, suggesting that performance became more similar to that of controls. Only the performance of patient IZS on two metrics changed statistical significance status post-treatment. In Semantic and Phonemic Fluency, patient IZS showed significantly different performance compared with healthy control pre-treatment, but post-treatment, his performance was not significantly different from healthy controls. In other words, the post-treatment performance was similar to that of healthy controls.

## 4. Discussion

The current study reports, for the first time, data on the effectiveness of tACS on EF in PPA, and it is the first study investigating the effects of tACS in PPA and in Greek-speaking individuals, in general. As a pilot, case-series investigation, the goal of this study was to establish the feasibility of the current treatment protocol to guide the design of a larger-scale clinical trial. The design of the treatment protocol relied on the hypothesis that the recruitment of EF processes, which are considered to be domain-general functions, can compensate, at least to some extent, for focal disruptions of specialized cognitive functions, such as language [28]. Using alpha-frequency tACS over the left DLPFC combined with behavioral treatment that targeted EF, we evaluated the extent to which language processing could also be improved, in addition to EF, by targeting EF functions in PPA.

The results showed numerical improvements both for EF and language functions across all four individuals, with the most prominent differences between pre- and post-treatment being those associated with EF. Despite the preliminary nature of this investigation, this was the expected pattern of results, given that both components (area of stimulation and behavioral treatment) of our treatment protocol targeted EF. Specifically, behavioral treatment focused on tasks that engage a variety of EF abilities, such as monitoring and attention, while the neuromodulation treatment targeted the left DLPFC, a brain area that has been extensively shown to support EF [7]. These results validate the effectiveness of the treatment protocol (i.e., improvements in the functions that are directly targeted) and provide the first evidence for the possible generalizability of these effects to functions that were not directly treated. It is also worth pointing out that patients who showed greater improvement in EF tended to exhibit numerical gains in language functions as well (as indexed by the number of metrics that showed changes from pre- to post-treatment). This pattern provides indications in favor of the hypothesis that improvement in domain-general functions may lead to at least partial improvement in more-domain-specific functions. As mentioned in the Introduction, in addition to their prominent language impairments, EF impairments are extensively reported in PPA [4,5,6]. Therefore, treatment protocols that target EF rather than language might be more beneficial for this population, as they could show effects across a wider variety of functions. Future studies that directly compare EF- and language-focused treatment protocols are needed to test this.

The language functions that showed numerical improvement varied across the four patients. While statistically significant improvements in language tasks were found only in one of the four patients (IZS), two language functions showed post-treatment numerical improvement in three out of the four patients: Semantic Fluency and Picture Description (percentage of nouns). In reality, both metrics evaluate semantic retrieval. In the Semantic Fluency task, the participant is asked to provide as many items as possible belonging to a given semantic category. In the Picture Description task, the participant is asked to freely describe what they see, which means that, once more, they need to retrieve words that are semantically related to the content of the picture. Semantic Fluency is a rather sensitive measure for the detection and classification of PPA and its variants [74,75]. The high sensitivity of this measure suggests that individuals with PPA really struggle with verbally producing semantically relevant words. These preliminary findings lay the groundwork for the development of treatments that are functionally relevant to the patients beyond the research setting.

Despite the semantic retrieval component of the Picture Description task, it is also important to highlight that this was the only task that allowed us to capture at least one aspect of daily communication abilities, i.e., narrative content. The numerical improvement in percentage of nouns across three out of the four patients provides some preliminary evidence that the proposed treatment might impact language functions in ways that are ecologically valid. However, given the lack of statistically significant effects, further research is required to assess the true extent of the real-world impact this type of treatment could have.

The only task in which all patients showed numerically improved performance after treatment was an EF task, the N-back task. The N-back task is widely used in neuropsychological assessments as an index of working memory, yet it is argued to capture other EF components as well, such as attention and monitoring [76,77]. Therefore, it could be speculated that the observed difference in N-back performance could reflect a broader EF improvement rather than an enhancement restricted to working memory. Our treatment protocol did not target a specific EF function; therefore, it is not feasible to determine the precise mechanism underlying this improvement.

Beyond the N-back task though, across the four cases, the pattern of improvement differed. In other words, not all individuals showed numerical improvement across the same tasks, and that was true both for the EF and the language tasks. A variety of reasons might give rise to these differences, such as the primary locus of atrophy, demographic variables (e.g., age), and overall cognitive status (as indexed, for example, with the MoCA). While large group studies might be able to control for the effects of these variables on the observed treatment effects, this information can also serve as guiding evidence for the design of future studies. For example, it might be more insightful to use a wide variety of assessment tasks and then calculate latent EF and language variables to index the shared variance across these domains. By doing so, one might be able to capture EF and language treatment effects without relying on specific tasks that not all individuals might show effects on.

Variability was also noted in the extent of responsiveness to treatment. Responsiveness to treatment might be driven by a number of factors, with a recent study showing that advanced age increases the variability in behavioral treatment effects after electrical stimulation [78]. In our sample, the youngest of our participants, IZS, showed the greatest improvement relative to the other cases across a variety of tasks. However, IZS also scored the highest among everyone on the MoCA and the MLSE. Therefore, one should keep in mind that the contribution of age to treatment responsiveness cannot be assessed independently of the contribution of their overall cognitive state at baseline. Future studies with larger sample sizes will need to add age as a covariate in the statistical analyses to investigate the contribution of these two variables independently of one another.

Contrary to most prior neuromodulation studies in PPA that have used tDCS, the current study used tACS for the first time. tACS allowed us to modulate activity at a specific frequency (i.e., the alpha band frequency), which has been previously implicated in EF [14,15,16]. tACS has been shown to have greater effects than tDCS in various EF processes across both individuals with cognitive impairments, such as MCI [25], and healthy adults [26,27]. However, unless we directly compare the effects of alpha-frequency tACS to tDCS, we will not be able to evaluate the significance of modulating activity at a specific frequency. Nonetheless, the current study provides evidence for the feasibility of tACS protocols in PPA and invites future research to investigate its effectiveness in a larger sham-controlled trial and with stimulation at other relevant frequency bands as well.

### Limitations

As a case series, the results of the present investigation have limited generalizability over the broader population. In other words, the findings might reflect patterns that are unique to these four individuals under investigation. The limited sample size also precludes multiple comparisons adjustments, which might lead to an increase in false positive rates. Hence, future group studies with larger samples and appropriate statistical corrections are needed to establish the effectiveness of the treatment protocol we used across individuals with PPA. Related to this issue is also the fact that sample heterogeneity might affect the conclusions we reach. The current sample included three individuals with lvPPA and one individual with svPPA, which does not allow us to investigate how treatment effects might differ across PPA variants and the underlying pathologies. Future investigations need to use variant-stratified designs to verify intervention effects across PPA variants. The lack of biomarker testing in two out of the four individuals also limits the interpretation of these findings with respect to the underlying pathology. Another important limitation is that we were not able to evaluate the added benefit of tACS stimulation over and above the benefits of the behavioral treatment alone. This is because all four individuals received behavioral treatment with active tACS without a second phase of behavioral treatment with sham stimulation or without a second group that would only receive behavioral treatment. However, given that prior studies have established the added benefit of neurostimulation across cognitive domains and populations, we expect that future studies with larger samples and a crossover, sham-controlled design will be able to shed light on this issue. Finally, the absence of long-term follow-up limits insight into the persistence of treatment effects. Future studies should include follow-up assessments of at least 3 months to evaluate effect duration.

## 5. Conclusions

This preliminary, case-series study showed that alpha-frequency tACS over the left DLPFC paired with behavioral treatment targeting EF improved EF as well as language processes in four Greek-speaking PPA individuals. While the patterns of improvement differed across the four individuals we studied, all of them showed greater changes in EF than in language abilities. This pattern is to be expected given that the treatment protocol specifically targeted EF. However, the fact that improvements—albeit less prominent—were also found for language processes provides evidence in support of the hypothesis that improvement in domain-general functions may lead to improvements in domain-specific functions as well. Given the small sample size, the results should be interpreted with caution. Nevertheless, they provide support and valuable guidance for the design of larger-scale, well-controlled clinical trials. Large clinical trials are needed to replicate the current findings and provide robust support for the application of this treatment protocol. Such studies will allow us to generalize conclusions to the broader PPA population and explore its application to other related disorders. Future studies should also examine the moderating effects of brain mechanisms and, in particular, of functional connectivity with respect to neuromodulation treatments that target EF and language outcomes.

## Figures and Tables

**Table 1:** Demographic characteristics and performance on Montreal Cognitive Assessment (MoCA) and Mini-Linguistic State Examination (MLSE) (total and subcomponents).

Participant	Age	Sex	Education	Years Since Onset	Diagnosis	MoCA
FAY	63	F	16	1	lvPPA	20
XTY	76	F	15	2	lvPPA	11
ADY	76	F	16	3	svPPA	15
IZS	60	M	18	1	lvPPA	21
	**MLSE** **Total**	**MLSE** **Articulation**	**MLSE** **Phonology**	**MLSE** **Semantics**	**MLSE** **Syntax**	**MLSE** **Working Memory**
FAY	88	30	29	17	7	5
XTY	73	30	24	8	7	4
ADY	75	30	27	8	6	4
IZS	96	30	29	19	8	10

**Table 2:** Individual executive functions and language performance per task. Raw scores are provided for pre- and post-treatment assessments, as well as their difference. In bold are tasks in which patients showed numerical improvement after treatment. Note: for TMT, a negative difference score is considered improvement as it shows a faster response.

			FAY			XTY			ADY			IZS		
Domain	Task	Metric	Pre	Post	Diff	Pre	Post	Diff	Pre	Post	Diff	Pre	Post	Diff
**Executiv** **e Function**	TMT-A	Seconds	93	88	**−5**	264	209	**−55**	69	80	11	61	61	0
	TMT-B	Seconds	300	300	0	300	300	0	300	300	0	216	195	**−21**
	Digit Span FW	Score	7	7	0	13	15	**2**	9	12	**3**	7	9	**2**
	Digit Span BW	Score	4	4	0	7	6	−1	8	12	**4**	7	9	**2**
	Spatial Span FW	Score	6	6	0	3	5	**2**	7	6	−1	5	7	**2**
	Spatial Span BW	Score	6	6	0	4	4	0	7	6	−1	5	8	**3**
	N-back	d-prime	1.25	2.21	**0.96**	0.12	1.84	**1.72**	2.71	4.38	**1.67**	3.37	3.43	**0.06**
**Language**	Spoken Naming	# of correct items	9	10	**1**	6	5	−1	1	1	0	26	28	**2**
	Picture Description	% nouns	0.17	0.10	−0.07	0.07	0.11	**0.04**	0.17	0.18	**0.01**	0.23	0.24	**0.01**
	Picture Description	% verbs	0.25	0.10	−0.15	0.17	0.18	**0.01**	0.20	0.15	−0.05	0.17	0.14	−0.03
	Semantic Fluency	# of items	5	8	**3**	5	7	**2**	11	6	−5	31	50	**19**
	Phonemic Fluency	# of items	4	4	0	15	22	**7**	9	5	−4	16	29	**13**

# = number; % = percentage out of total number of words produced.

**Table 3:** Cohen’s d and statistical significance results of Crawford–Garthwaite Bayesian tests (*p*-values) per task, per patient, for pre- and post-treatment performance. *: *p* < 0.05; ~: *p* < 0.10; NS = non-significant.

Domain	Task	Patient	Pre		Post	
			Cohen’s d	*p*-Value	Cohen’s d	*p*-Value
Executive Function	TMT-A	FAY	8.86	<0.001 *	8.14	<0.001 *
		XTY	14.51	<0.001 *	10.92	<0.001 *
		ADY	1.78	0.047 *	2.50	0.011 *
		IZS	4.26	<0.001 *	4.26	<0.001 *
	TMT-B	FAY	12.66	<0.001 *	12.66	<0.001 *
		XTY	4.51	<0.001 *	4.51	<0.001 *
		ADY	4.51	<0.001 *	4.51	<0.001 *
		IZS	8.15	<0.001 *	7.02	<0.001 *
	N-back	FAY	−2.95	0.005 *	−1.80	0.047 *
		XTY	−4.31	<0.001 *	−2.25	0.020 *
		ADY	−1.20	NS	0.81	NS
		IZS	−0.41	NS	−0.33	NS
Language	Spoken Naming	FAY	−6.86	<0.001 *	−6.46	<0.001 *
		XTY	−8.08	<0.001 *	−8.48	<0.001 *
		ADY	−10.09	<0.001 *	−10.09	<0.001 *
		IZS	0.00	NS	0.81	NS
	Picture Description—Nouns	FAY	−1.18	NS	−2.56	0.009 *
		XTY	−3.08	0.003 *	−2.30	0.016 *
		ADY	−1.08	NS	−0.93	NS
		IZS	0.08	NS	0.23	NS
	Picture Description—Verbs	FAY	0.80	NS	−1.70	0.054 ~
		XTY	−0.45	NS	−0.30	NS
		ADY	0.04	NS	−0.75	NS
		IZS	−0.50	NS	−0.97	NS
	Semantic Fluency	FAY	−4.92	<0.001 *	−4.62	<0.001 *
		XTY	−4.92	<0.001 *	−4.72	<0.001 *
		ADY	−4.33	<0.001 *	−4.82	<0.001 *
		IZS	−2.35	0.017 *	−0.47	NS
	Phonemic Fluency	FAY	−4.07	<0.001 *	−4.07	<0.001 *
		XTY	−2.75	0.008 *	−1.90	0.040 *
		ADY	−3.47	0.002 *	−3.95	0.001 *
		IZS	−2.63	0.010 *	−1.06	NS

## Data Availability

The original contributions presented in this study are included in the article. Further inquiries can be directed to the corresponding author.

## Supplementary Materials

The following supporting information can be downloaded at https://www.mdpi.com/article/10.3390/brainsci15111199/s1: Table S1: Descriptive statistics for healthy control groups.
