# Supplementary material for: Targeting Executive Function and Language Impairments with tACS Combined with Behavioral Intervention in Primary Progressive Aphasia: A Case-Series, Pilot Investigation
Source: Brain Sci. 2025 Nov 7;15(11):1199. doi: 10.3390/brainsci15111199 (PMC12650604; doi:10.3390/brainsci15111199)
Supplement: Supplementary file 1 [file brainsci-15-01199-s001.zip › brainsci-3975005-supplementary.pdf]

Supplementary Material

Table S1. Descriptive statistics (mean and standard deviation) for Age and Education for the healthy control (HC) groups used to compare patients' performance, per task. Note that for TMT two difference HC groups were used to match patients' ages.

Domain	***Task***	***Patient***	***HC Age Mean (SD)***	***HC Education Mean (SD)***	***HC N***
Executive Function	TMT	FAY	61.94 (1.50)	15.45 (1.31)	31
		XTY	77.21 (1.49)	15.29 (1.80)	32
		ADY	77.21 (1.49)	15.29 (1.80)	32
		IZS	61.94 (1.50)	15.45 (1.31)	31
	N-back	FAY<br>XTY<br>ADY<br>IZS	72.19 (5.98)	17.76 (3.31)	21
Language	Spoken Naming	FAY<br>XTY<br>ADY<br>IZS	62.45 (4.88)	14.62 (2.98)	35
	Picture Description	FAY<br>XTY<br>ADY<br>IZS	56.11 (4.80)	14.67 (3.82)	27
	Phonemic/Semantic Fluency	FAY<br>XTY<br>ADY<br>IZS	65.60 (4.78)	16 (1.65)	15
